# Trends in coordination of rhenium organometallic complexes in the Protein Data Bank

**DOI:** 10.1107/S2052252522000665

**Published:** 2022-02-25

**Authors:** Alice Brink, Francois J. F. Jacobs, John R. Helliwell

**Affiliations:** a Chemistry Department, University of the Free State, Nelson Mandela Drive, Bloemfontein, South Africa; b Department of Chemistry, The University of Manchester, Oxford Road, Manchester, United Kingdom

**Keywords:** organometallic complexes, proteins, rhenium, technetium, radiopharmaceuticals, radioisotopes, transition metals

## Abstract

Radiopharmaceutical development has similar overall characteristics to any biomedical drug development requiring a compound’s stability, aqueous solubility and selectivity to a specific disease site. However, organometallic complexes containing ^188/186^Re or ^99m^Tc involve a *d*-block transition-metal radioactive isotope and therefore bring additional factors such as metal oxidation states, isotope purity and half life into play. This topical review is focused on the development of radiopharmaceuticals containing the radioisotopes of rhenium and technetium and, therefore, on the occurrence of these organometallic complexes in protein structures in the Worldwide Protein Data Bank (wwPDB). The purpose of incorporating the group 7 transition metals of rhenium/technetium in the protein and the reasons for study by protein crystallography are described, as certain PDB studies were not aimed at drug development. Technetium is used as a medical diagnostic agent and involves the ^99m^Tc isotope which decays to release gamma radiation, thereby employed for its use in gamma imaging. Due to the periodic relationship among group 7 transition metals, the coordination chemistry of rhenium is similar (but not identical) to that of technetium. The types of reactions the potential model radiopharmaceutical would prefer to partake in, and by extension knowing which proteins and biomolecules the compound would react with *in vivo*, are needed. Crystallography studies, both small molecule and macromolecular, are a key aspect in understanding chemical coordination. Analyses of bonding modes, coordination to particular residues and crystallization conditions are presented. In our Forward look as a concluding summary of this topical review, the question we ask is: what is the best way for this field to progress?

## Abstract 2

This topical review is focused on the development of radiopharmaceuticals containing the radioisotopes of rhenium and technetium, and examines the occurrence of these transition-metal complexes in protein structures in the Worldwide Protein Data Bank. In summing up and looking forward, the authors ask what is the best way for this field to progress.

## 1. Introduction

Drug development is a complex study involving multiple factors. The development of one new medical product, from its discovery to the time it is made available for the treatment of patients, takes on average 10–15 years, with an average cost of $800 million to $1.6 billion to research and develop each successful drug. For every drug that receives approval, an estimated 10 000 compounds have entered the research and development pipeline and been discarded for particular reasons (PhRMA, 2015 ▸; Eckstein, 2005 ▸; Tonkens, 2005 ▸; Torjesen, 2015 ▸). Research typically selects a ‘target’ for a potential medicine, which is generally a single molecule (*e.g.* a gene or protein) that is involved in a particular disease. It is important to confirm how a chosen target is involved in the disease and whether it can interact and be affected by a drug molecule. The search for a promising molecule or ‘lead compound’ can now be undertaken with the hope that the disease can be arrested. Lead compounds are assessed early on for safety according to their pharmokinetics or absorption, distribution, metabolism, excretion and toxicity (ADME/Tox) properties, whereby the function and performance of hundreds of different compounds are tested *in vitro* (Ruiz-Garcia *et al.*, 2008 ▸; Tuntland *et al.*, 2014 ▸; Chung *et al.*, 2015 ▸). Due to the existence of libraries of compounds with known pharmacokinetic properties, it is possible to generate predictive models through machine-learning techniques. This has been successfully employed in pharmacokinetic studies and is helping the complex process of designing new drug candidates from the use of reliable machine-learning models and from studies of quantitative structure–activity relationships (Maltarollo *et al.*, 2015 ▸).

Traditionally, medicinal chemistry has focused on organic, not inorganic, chemistry. The potential role of organometallic complexes, relatively speaking, has been neglected. The inclusion of metal atoms significantly increases the variety of building blocks which can be made, but at the same time increases the complexity of mechanistic behaviour, protein coordination, stability *etc.*, hence the reason for their minority within the drug market (Nogrady & Weaver, 2005 ▸). In radiopharmaceutical development, whereby the model complexes contain a radioactive isotope, additional factors must be considered such as isotope purity, half life, the cost and availability of the isotope as well as radiation dose (Liu, 2004 ▸). Our interest is in the development of radiopharmaceuticals containing the radioisotopes of technetium and rhenium. Technetium in the form of ^99m^Tc decays to release gamma radiation and is thereby employed for its use in gamma or single photon emission computed tomography (SPECT) imaging. It is widely utilized for diagnostic nuclear medicine with 80% of current radiopharmaceuticals administered clinically containing this radioisotope (Liu, 2004 ▸; Kluba & Mindt, 2013 ▸). The ^99m^Tc isotope is routinely used in brain (Jurisson *et al.*, 1993 ▸; Dilworth & Parrott, 1998 ▸), heart (Holman *et al.*, 1984 ▸; Gerson *et al.*, 1983 ▸), bone (Dilworth & Parrott, 1998 ▸) and thyroid (Dodds & Powell, 1968 ▸) imaging. Moreover, the isotope is being investigated for selective cancer imaging and multidrug resistance (Piwnica-Worms *et al.*, 1995 ▸; Herman *et al.*, 1995 ▸; Goffin *et al.*, 2017 ▸; Monteiro *et al.*, 2017 ▸; Lin *et al.*, 2018 ▸). Due to the chemical periodic relationship among group 7 transition metals, the coordination chemistry of rhenium is similar (but not identical) to that of technetium. This similarity is advantageous as it allows bifunctional chelating ligands that have been developed for ^99m^Tc to be used for rhenium and *vice versa*. The advantage of working first with rhenium is that experimental synthesis can be conducted on a non-radioactive ‘cold’ isotope (whereas all technetium isotopes are radioactive), and thus permits detailed chemical and reactivity analysis without the hazards of using the radioactive isotopes ^186/188^Re, ^99m^Tc and ^99^Tc. There are two rhenium radionuclides utilized in therapeutic nuclear medicine, namely ^186^Re and ^188^Re, which function by means of β-irradiation. Rhenium complexes have been developed for bone metastasis (Lam *et al.*, 2009 ▸; Liepe *et al.*, 2005 ▸), liver cancer (Lambert *et al.*, 2005 ▸) and as steroid mimics (Chi & Katzenellenbogen, 1993 ▸; Chi *et al.*, 1994 ▸; DiZio *et al.*, 1992 ▸). Investigating the fundamental chemical behaviour of a given chemical complex is necessary to predict what types of reactions the potential model radiopharmaceutical would partake in and, by extension, know which amino acids in proteins and biomolecules the compound would react with *in vivo*. These structure–function–reactivity studies, both within small-molecule and macromolecular research fields, are a key aspect to predicting and/or optimizing chemical coordination. These properties are utilized in the field of fragment-based drug development and high-throughput screening (Erlanson, 2012 ▸; Murray *et al.*, 2012 ▸; Joseph-McCarthy *et al.*, 2014 ▸).

The various differences between small-molecule and macromolecular crystallography methods, software and results often inhibit cross utilization, *i.e.* the interoperability of the data (Brink & Helliwell, 2019*b* ▸). New software-usage trends, such as with *GOLD* (Jones *et al.*, 1997 ▸) or *CSD-CrossMiner*, both developed by the Cambridge Crystallographic Data Centre (CCDC) (Groom *et al.*, 2016 ▸), are improving this interdisciplinary and interoperability data usage. This review focuses on protein structures found in the Protein Data Bank (PDB) that contain a rhenium or technetium metal centre. Our aims are to better understand:

(*a*) the chemical basis of this transition-metal family in its interactions with biological molecules;

(*b*) the effects that a non-natural metal may have within an organic macromolecular model;

(*c*) whether any possible chemical trends can be identified from the biological structural data; and

(*d*) the likely stability or even strict relevance of the measured structural data, such as that involving the specific crystallization conditions used and tabulated in Table 1 ▸. As a simple question we ask: does the pH used for crystallization match the pH values seen in the human body? For example, stomach acid pH is ∼1 and the pH of the blood is 7.4. Many protein crystals are grown according to the general principles of macromolecular crystallization and crystal perfection, optimized by technique and technologies to measure the best possible diffraction data as described in Chayen *et al.* (2010 ▸). As Table 1 ▸ documents, a good fraction of the crystals are grown at or around pH 7. There are none for pH 1 however, *i.e.* the relevant pH if a compound was to be administered orally and therefore should be chemically stable within the stomach.

In our *Forward look* as a concluding summary of this review, the question we ask is: what is the best way for this field to progress? Several chemical mechanistic studies have focused on protein–metal coordination and selectivity for rhenium (Zobi & Spingler, 2012 ▸; Santoro *et al.*, 2012 ▸; Takematsu *et al.*, 2013 ▸; Brink & Helliwell, 2019*a* ▸, 2017 ▸; Binkley *et al.*, 2011 ▸), for platinum (Messori & Merlino, 2016 ▸; Tanley *et al.*, 2014 ▸; Wang *et al.*, 2017 ▸) or for rhodium (Loreto *et al.*, 2021 ▸; Abe *et al.*, 2009 ▸; Daubit *et al.*, 2020 ▸). However, protein–ligand (*i.e.* the organic ligand bound to the organometallic complex) interactions may too play a role. Each of these mechanisms will have a direct effect on the viability of the complex as a radiopharmaceutical and on the design of the next iteration of potential model radiopharmaceuticals. Theoretical calculations describing the reactivity binding of rhenium complexes to biomolecules (Oliveira *et al.*, 2013 ▸; Aliyan *et al.*, 2017 ▸; Carreño *et al.*, 2021 ▸), as well as continued structural evaluations of future PDB entries, would indicate if there are new trends forming a mechanistic driving-force preferential for either protein–metal or protein–ligand coordination.

## 2. Overview of the purposes of the depositors of these crystal structures

Table 1 ▸ presents all the rhenium-bound protein crystal structures in the wwPDB including key crystallographic and synthetic aspects. A list of amino-acid residues directly bound to the rhenium metal centre as well as weak interactions from the protein to the organic ligand of the organometallic complex are specified. Where the structure factors were made available we have also examined any uninterpreted residual-difference electron-density features and offer appropriate comments. We also include the crystallization conditions used for each PDB entry.

The purposes of the protein studies listed in the wwPDB that contain rhenium metal centres show a variety of applications. These have included (i) multi- or single-wavelength anomalous dispersion (MAD or SAD) phasing using relatively simple rhenium compounds, (ii) electron transfer and/or electron tunnelling or (iii) for medical applications. We describe all three aspects and highlight key observations made either by the authors or through our examination of the data. Many studies have included the investigation of both rhenium and technetium with their respective biological activity and confirmed the presence of technetium via alternative methods of characterization (*i.e.* NMR, infra-red spectroscopy or mass spectrometry). However, the protein structures currently available in the PDB only include rhenium, not technetium.

### 2.1. Rhenium for MAD or SAD phasing

**Figure 1:**
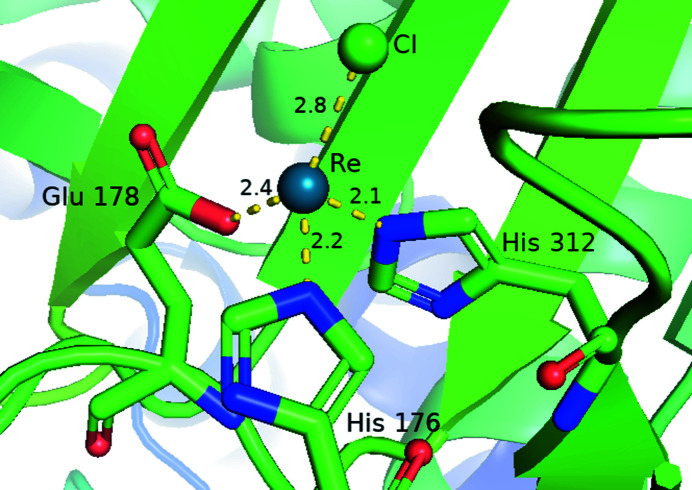
A coordination-site view of 6f9p at one of the four rhenium sites. The other sites are equivalent to the one shown. This figure has been made using *PyMOL* (DeLano, 2002 ▸). In this and all figures carbon atoms are indicated as green (as are the ribbon diagrams for protein structures), nitro­gens are blue, oxygens are red, sulfur is yellow, rheniums are cyan, copper is orange and chlorine is dark green. Hydrogen atoms are generally omitted due to the lower-resolution data typically obtained by macromolecular crystallographic investigations; however, in this topical review, when their positions are more accurately known, such as in the case of 1b0q, hydrogen atoms are coloured light grey. The distances shown are in Å.

The purpose of rhenium in these protein structures was for MAD or SAD phasing and not the medical application of the metal’s effects. However, that said, within this review’s objective of extracting coordination data, these protein structures make a valuable contribution to possible trends for preferential binding sites and thereby expand the medical-application potential. The protein structures (with PDB codes and citations given in parentheses) that involved rhenium for phasing used relatively simple rhenium compounds: [ReCl_6_]^2−^ (3lya, Eichinger *et al.*, 2011 ▸; 6f9p, Bastard *et al.*, 2018 ▸), perrhenate [ReO_4_]^−^ (1hnu, Mursula *et al.* 2001 ▸; 1k4j, Watson *et al.*, 2002 ▸) or *fac*-[Re(CO)_3_]^+^ (5k1j, Ciccone *et al.*, 2016 ▸), all of which are commercially available starting complexes and are readily soluble in water. Hence, interest in the specific chemistry of rhenium (*i.e.* oxidation states, coordination, stability *etc.*) was not considered a research priority of these particular studies. Variation in protein–metal coordination is found across this group, such as for 1hnu where rhenium is bound to the active site of the enoyl-CoA isomerase. Structure 5k1j has rhenium coordinated at His88, as well as being present at multiple sites in varying low occupancy. The study 3lya altogether shows 16 bound [ReCl_6_]^2−^ ions to residues including histidine, tryptophan, aspartic and glutamic acid. The study 6f9p, also utilizing [ReCl_6_]^2−^, found that the rhenium retains only one chloride and its ligands are replaced by amino-acid interactions, notably two histidines, *i.e.* a special case for this particular protein (Fig. 1 ▸).

### 2.2. Rhenium-based crystal structures for studying electron transfer and/or tunnelling

**Figure 2:**
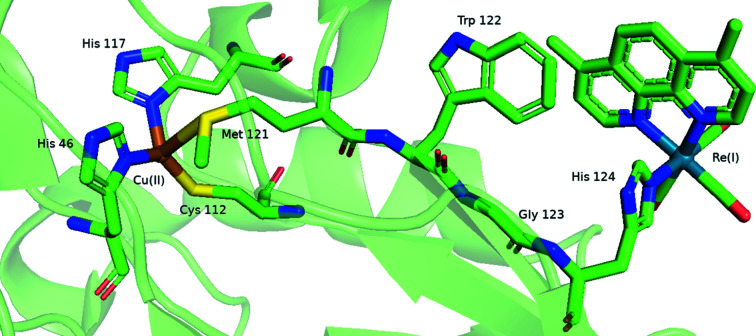
A coordination-site view of 2i7o indicating both the coordination of a rhenium(I) tri­carbonyl complex, *fac*-[Re^I^(CO)_3_], via the histidine and the Cu^II^ to the azurin protein. This figure has been created with *PyMOL*.

Electron tunnelling is a quantum mechanical phenomenon that occurs when electrons move through a barrier that classically should not be possible to traverse. In proteins, electron tunnelling can move electrons between donor and acceptor sites separated by distances ranging from 10 to 30 Å on a millisecond or even femtosecond time scale (Stuchebrukhov, 2010 ▸; Tezcan *et al.*, 2001 ▸). As an example, protein structures 2i7o (Shih *et al.*, 2008 ▸), 6mjs, 6mjt and 6mjr (Takematsu *et al.*, 2019 ▸) reported the use of a rhenium(I) tri­carbonyl complex, *fac*-[Re^I^(CO)_3_], in a mutant *Pseudomonas aeruginosa* azurin to examine the electron-transfer capability between distant metal redox centres within the protein. In 2i7o the rhenium complex [Re^I^(CO)_3_(dmp)] (dmp = 4,7-di­methyl-1,10-phenanthroline) was attached to the histidine-124 residue (Fig. 2 ▸). The 1.5 Å resolution crystal structure of the Re-labelled protein shows that the ligand (dmp) and the trypophan-122 indole group are near van der Waals interaction distances (∼4 Å), and the Cu–Re distance is 19.4 Å. Structures 6mjs, 6mjt and 6mjr are coordinated to the His126 via the imidazole N ring bonded to the octahedral *fac*-[Re(CO)_3_]^+^ moiety in the sixth ligand position. Additional studies [1i53, Di Bilio *et al.* (2001 ▸); 1jzi, Crane *et al.* (2001 ▸); 1r1c, Miller *et al.* (2003 ▸); 2fnw, Blanco-Rodríguez *et al.* (2006 ▸); 4k9j, Takematsu *et al.* (2013 ▸); 3ibo, Blanco-Rodríguez *et al.* (2006 ▸); 2i7s, Blanco-Rodríguez *et al.* (2009 ▸)] similarly show the direct coordination of the *fac*-[Re^I^(CO)_3_] core to the protein via the histidine imidazole moiety.

### 2.3. Medical applications

Technetium and rhenium can exist in a range of oxidation states ranging from +7 to −1 (rhenium can range further to −3). Due to technetium’s (^99m^Tc) ideal radiodiagnostic properties (*i.e.* a half life of 6.02 h, gamma radiation of 141 keV and sourced from a ^99^Mo–^99m^Tc generator) (Firestone *et al.*, 1996 ▸; Boswell & Brechbiel, 2007 ▸), as well as rhenium’s similarity in chemistry, including its own ^188/186^Re isotope used for therapy, these elements have been extensively investigated for medical applications. Multiple generations of complexes have been developed utilizing various oxidation states and cores, such as pertechnetate, ^99m^TcO_4_^−^ (in the +7 oxidation state and commercially available as TechneLite) (Dodds & Powell, 1968 ▸), and the Tc^+5^ mono-oxo (Tc=O) core (Ceretec) (Mazzi *et al.*, 2007 ▸). The ^99m^Tc-MDP also known as ^99m^Tc-medronate (Osteolite) used for imaging of bone metastasis has a +4 oxidation state and is thought to coordinate in an octahedral fashion. Furthermore, ^99m^Tc-tetrofosmin (Myoview) (Kelly *et al.*, 1993 ▸) is a cationic compound and is used in myocardial perfusion imaging. It contains a Tc^+5^ *trans* di-oxo (Tc=O_2_) core. Moreover, ^99m^Tc-NOET, a neutrally charged myocardial imaging agent, consists of a ^99m^Tc(V)N^2+^ core (Pasqualini *et al.*, 1994 ▸). In addition, ^99m^Tc-sestamibi (Cardiolite) has a +1 oxidation state and has octahedral coordination surrounded by iso­nitrile ligands. The water-soluble and readily synthesized *fac*-[Tc^99m^(CO)_3_]^+^ core from the IsoLink Kit (supplied by Mallinckrodt) similarly has a +1 oxidation state with octahedral coordination (Alberto *et al.*, 1999 ▸, 2001 ▸; Schibli *et al.*, 2000 ▸).

The oxidation states and transition-metal core play a key role in synthesis and solubility, as well as the general and biological chemistry (Liu, 2004 ▸; Alberto *et al.*, 2020 ▸). We have therefore grouped and described the following medically applicable PDB structures according to the Re/Tc metal core which is found in these structures.

*Caution*: ^99^Tc is a β^−^ emitter with a half life of *ca* 210 000 years, ^99m^Tc is a γ emitter with a half life of *ca* 6 h, and ^186^Re and ^188^Re are β^−^ emitters with half lives of *ca* 3.7 d and 17 h, respectively. Thus, all experiments have to be performed in laboratories approved for working with low-level radioactive materials. Naturally occurring rhenium, _75_Re, is 37.4% ^185^Re (considered observably stable) and 62.6% ^187^Re (an unstable isotope but it has a very long half life of *ca* 10^10^ years), it is therefore considered stable for standard laboratory use.

#### 2.3.1. Radiopharmaceutical development utilizing rhenium oxo (Re/Tc^V^O core) complex coordination to proteins

**Figure 3:**
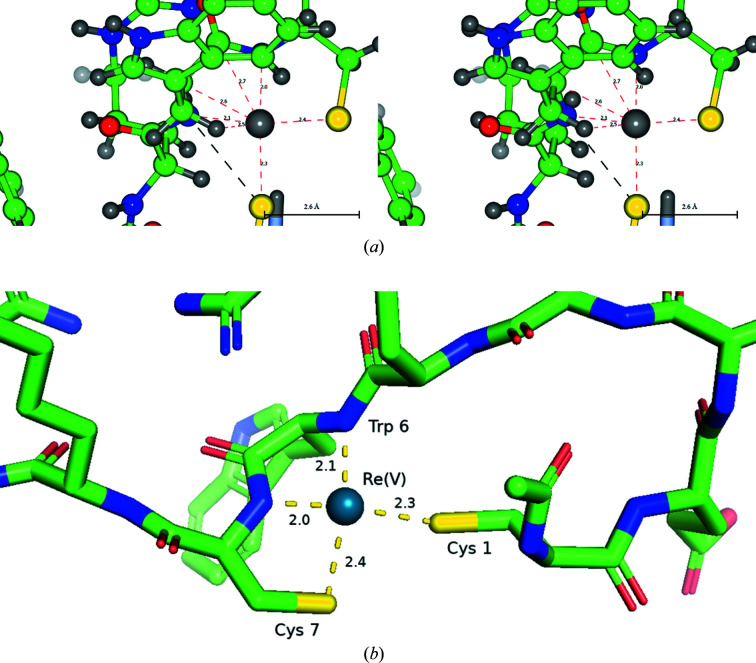
(*a*) A stereoview of 1b0q at the rhenium binding site. This part of the figure was created using *CCP*4*mg* (McNicholas *et al.*, 2011 ▸). The distances shown are in Å. (*b*) The binding site of 1b0q showing the cyclic coordination of Re(V) to the α-MSH analog. This part of the figure was created using *PyMOL*. The distances shown are in Å.

Radiopharmaceutical development utilizing rhenium-188 and technetium-99m metal coordination to proteins has been investigated by Giblin *et al.* (1998 ▸) utilizing the Re/Tc^V^ oxo core. The NMR study investigated the coordination of a [ReOCl_3_(Me_2_S)(OPPh_3_)] complex in solution. The authors’ goal was to design ^188^Re- or ^99m^Tc-radiolabelled α-melanocyte stimulating hormone (α-MSH) analogues in which metal coordination was an integral part of the molecule’s structure (Fig. 3 ▸).

Both the Tc and Re oxo complexes in 1b0q (Giblin *et al.*, 1998 ▸) were in the +5 oxidation state, which tends to prefer a square pyramidal coordination geometry. The cyclic Re–peptide analogue, ReMSH, was synthesized by incorporating the Re^V^O core into APOMSH via trans chelation from the [ReOCl_3_(Me_2_S)(OPPh_3_)] organometallic complex. The α-MSH analogues, cyclized through site-specific rhenium and technetium metal coordination, were structurally characterized and analysed for their ability to bind to α-MSH receptors present on melanoma cells and in tumour-bearing mice. Crystal structure analysis of the Re–peptide complex showed that the disulfide bond of the original peptide was replaced by thiol­ate–metal–thiol­ate cyclization. When the metal binding site was redesigned, a second-generation Re–peptide complex (ReCCMSH) formed, which displayed a receptor binding affinity of 2.9 n*M*, 25-fold higher than the initial ReMSH analogue.

#### 2.3.2. Protein coordination with perrhenate oxo cores involving molybdate substitution in molybdate-binding periplasmic protein

**Figure 4:**
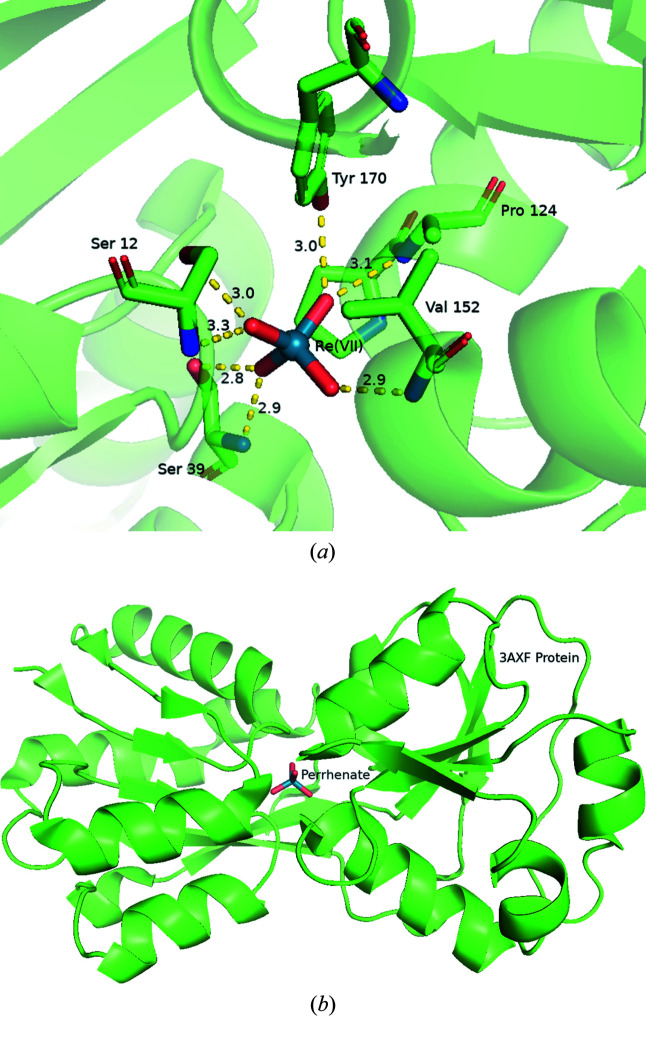
(*a*) A detailed view of the multiple weak interactions occurring between perrhenate and the protein residues of 3axf. This was made using *PyMOL*. The distances shown are in Å. (*b*) A distant view of the perrhenate situated overall in the protein.

The protein coordination to an alternative rhenium core was explored by Aryal *et al.* (2012 ▸) (3axf) who presented a strategy to engineer proteins that may selectively recognize the perrhenate (ReO_4_^−^) ion so as to develop a new method to label proteins. The ReO_4_^−^ anion is tetrahedral in shape and contains the rhenium atom in the +7 oxidation state with a *d* ^0^ configuration. It is similar in size and shape to perchlorate and the valence is isoelectronic to permanganate. It is also stable over a broad pH range (Eiroa-Lledo *et al.*, 2020 ▸). The chemistry of the perrhenate ion is like that of the pertechnetate ion ^99m/99^TcO_4_^−^, which again makes it ideal for exploratory research without having to utilize the radioactive ^99m/99^Tc radionuclide (Mazzi *et al.*, 2007 ▸). The authors determined that the molybdate (MoO_4_^2−^) binding protein (ModA) from *Escherichia coli* can bind perrhenate with high affinity and were able to solve the crystal structure of ModA with a bound ReO_4_^−^ (3r26). The authors also synthesized a mutant protein containing a disulfide linkage, which exhibited increased affinity for the perrhenate (3axf). These protein structures both indicate that the ReO_4_^−^ ion occupies the MoO_4_^2−^ binding site using the same amino-acid residues that are involved in molybdate binding. The overall protein structure of the perrhenate-bound ModA is unchanged compared with that of the molybdate-bound form (see Fig. 4 ▸).

The affinity of most proteins for the radionuclides of rhenium and technetium is not known. The effect of the bifunctional chelator on the metal reactivity (Jacobs *et al.*, 2021 ▸; Brink *et al.*, 2014 ▸; Schutte *et al.*, 2011 ▸, 2012 ▸) and the stabilities of the bidentate [2+1] *N,N′*; *N,O′*; *O,O′* or tridentate coordinated complexes under physiological conditions are still being explored (Schibli *et al.*, 2000 ▸; Schibli & Schubiger, 2002 ▸). These studies (3axf and 3r26) therefore make a valuable contribution to understanding molybdate protein interactions, particularly if it can be generalized so that more perrhenate-bound proteins can selectively be stabilized with the presence of disulfide linkages. The authors indicate that the binding protein originates from a bacterium as the molybdate transporter in *Homo sapiens* has yet to be discovered. This could be applied for targeted delivery to an organ of concern, if other molybdate/perrhenate-labelled proteins could be identified. Secondly, the question arises if it would be possible to substitute the perrhenate oxo core (ReO_4_^−^) with an alternative core, such as the *fac*-[Re(CO)_3_]^+^ core that we have shown in our studies, summarized in Section 2.3.3, to be able to coordinate to multiple types of amino acids. This would increase the absorption and the clinical X-ray contrast.

#### 2.3.3. Rhenium–protein coordination utilizing the *fac*-[Re^I^(CO)_3_] core

The tri­carbonyl cores of rhenium and technetium, *fac*-[*M* ^I^(CO)_3_] (*M* = Re or Tc), are widely utilized due to their water solubility, as well as their relatively simple synthetic procedure which is conducted under mild conditions in aqueous solutions. The aqua complex *fac*-[*M* ^I^(CO)_3_(H_2_O)_3_] is coordinated by three tightly bound CO ligands and three labilizable water ligands. It is a highly attractive possibility for radiopharmaceutical design due to the high stability of the *fac*-[*M*(CO)_3_]^+^ core in water and the potential of exchanging the labile solvent ligands to allow coordination with many different types of ligands (Alberto *et al.*, 1999 ▸, 2001 ▸; Schibli *et al.*, 2000 ▸; Jacobs *et al.*, 2021 ▸).

Within the wwPDB, 3rj7 (Can *et al.*, 2012 ▸) describes rhenium bio-organometallic carbonic anhydrase inhibitors (CAI) with nanomolar affinities for specific CA subtypes. CAs are targets for cancer diagnosis and therapy because of hypoxia-induced overexpression of hCAIX and hCAXII (hCA = human CA) in several malignancies, including cancer (Lindskog, 1997 ▸; Supuran, 2008*a* ▸,*b* ▸; Bose & Satyanarayana, 2017 ▸). In 3rj7, the study included both rhenium and technetium-99m aryl­sulfonamide, sulfamide, and sulfamate-based CAIs containing the [(Cp–R)*M*(CO)_3_] complex (*M* = Re or ^99m^Tc; Cp = cyclo­pentadienyl) (Can *et al.*, 2012 ▸). All these complexes were in the +1 oxidation state and octahedral coordination. The [(Cp–R)Re(CO)_3_] complex is found in the binding pocket of hCAII with no covalent bonds formed between the protein and the Re metal centre. However, the deprotonated nitro­gen of the aryl­sulfonamide terminus of the [(Cp–R)Re(CO)_3_] complex coordinates to the Zn atom in the active site, thus forming a protein–ligand bond. The [(Cp)Re(CO)_3_] complex has no further interactions with either the protein or water molecules (Can *et al.*, 2012 ▸). However, there are hydrophobic interactions between the [(Cp)Re(CO)_3_] moiety and the hydro­phobic parts of Phe131, Leu198 and Pro202 (RCSB NGL ligand viewer https://www.rcsb.org/docs/3d-viewers/ngl#ligand-viewer-options) (Rose & Hildebrand, 2015 ▸; Rose *et al.*, 2017 ▸).

**Figure 5:**
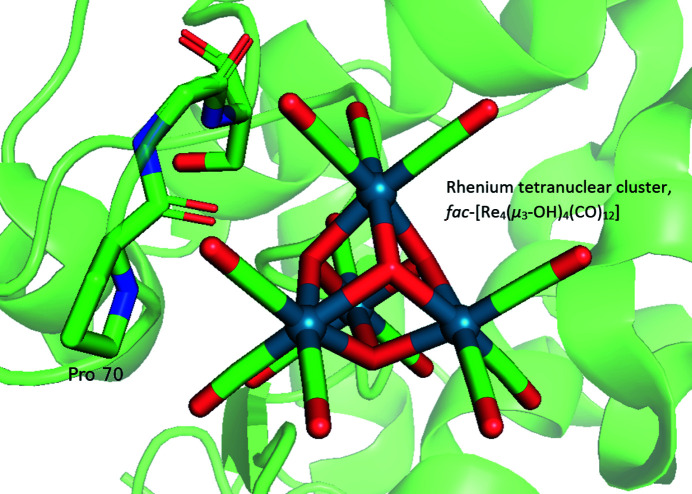
A *PyMOL* view of the rhenium tetranuclear cluster, *fac*-[Re_4_(μ_3_-OH)_4_(CO)_12_], in the vicinity of Pro70 residue in the protein structure 6ro3.

Other studies that have examined the coordination of *fac*-[Re^I^(CO)_3_] complexes to proteins, specifically to understand the protein–metal coordination for radiopharmaceutical development, have been described by Binkley *et al.* (2010 ▸) (3kam), Binkley *et al.* (2011 ▸), Zobi & Spingler (2012 ▸) (3qng, 3qe8) and Santoro *et al.* (2012 ▸). The studies have shown that rhenium–protein coordination utilizing the *fac*-[Re^I^(CO)_3_] core has consistencies, such as the metal core showing binding preference to a histidine imidazole [Binkley *et al.*, 2011 ▸; Zobi & Spingler, 2012 ▸; Santoro *et al.*, 2012 ▸; Takematsu *et al.*, 2013 ▸ (structure 4k9j studied for interest in electron tunnelling)]. The exception to the histidine imidazole sole preference was our study (Brink & Helliwell, 2017 ▸), which employed two X-ray wavelengths for rhenium resonant-scattering signal enhancement and enabled the finding of rhenium transition-metal placements, even at low occupancy. With that approach, rhenium coordination was also observed in binding to aspartic acid, glutamic acid, arginine and leucine residues (5nbj; Brink & Helliwell, 2017 ▸). The kinetic formation of tetranuclear rhenium clusters appropriate for theranostic applications, albeit in the crystal and rather slow (up to two years), has also been observed with the *fac*-[Re^I^(CO)_3_] core (6ro3, 6ro5; Brink & Helliwell, 2019*a* ▸) (see Fig. 5 ▸).

**Figure 6:**
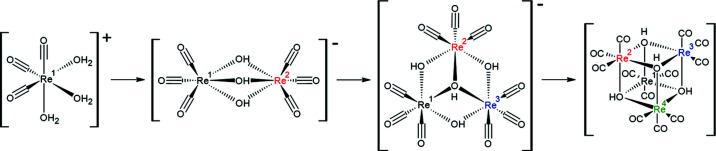
A schematic representation of the formation of the dinuclear, trinuclear and tetranuclear rhenium clusters starting from the *fac*-[Re(CO)_3_]^+^ core. Each rhenium atom is coloured individually according to stepwise incorporation into the cluster; on its own at far left it is black, then second from left the added rhenium is red and so on for the third (blue) from left and the fourth one (green) at far right.

Fig. 6 ▸ summarizes the complete kinetic stepwise formation that the rhenium complexes can undergo, and where the mono- and tetranuclear complexes {*fac*-[Re(CO)_3_]^+^ and *fac*-[Re_4_(μ_3_-OH)_4_(CO)_12_]} were observed in the protein–rhenium crystal structures studied. We deem this expanded group of rhenium complexes seen bound to a protein as a breakthrough in the whole field, particularly as it is synthetically possible to substitute one rhenium atom with either technetium-99m or technetium-99 to form a mixed rhenium and technetium version where more than one metal centre is present and with possible further theranostic applications (Mokolokolo *et al.*, 2018 ▸; Frei *et al.*, 2018 ▸).

Of additional medical interest is the recent report of the synthesis and biophysical evaluation of a series of *fac*-[Re^I^(CO)_3_(bipy)]^+^ (bipy = 2,2 bi­pyridine ligand) complexes as inhibitors of the SARS-CoV-2 main protease 3CL^pro^ (3-chymotrypsin-like protease) (Karges *et al.*, 2021 ▸). Mass-spectrometry experiments verified the covalent binding of a single [Re^I^(CO)_3_] complex to the 3CL^pro^ preferentially via the Cys145 amino acid. The authors suggest that rhenium(I) tri­carbonyl complexes can serve as a starting scaffold for the development of potent selective SARS-CoV-2 inhibitors.

## 3. Forward look: what is the best way for this field to progress?

We have stated the need to identify any possible trends that may be occurring in this field as they could provide key information on whether there is any binding preference occurring between the group 7 transition-metal series and proteins. The themes of other research labs have been described and particularly the reasons as to why rhenium was chosen. It is also important to note the variety of proteins used, the wide range of organometallic complexes and the crystallization conditions (Table 1 ▸).

In our research, we have firstly identified that there are more amino-acid types binding to rhenium organometallics than previously seen. Secondly, via our most recent research, we have expanded the available repertoire of rhenium compounds to include multi-metal-centre complexes (Brink & Helliwell, 2017 ▸, 2019*a* ▸). Both these advances have the potential to increase the absorption of the organometallic complex at the organ being imaged. These are promising steps forward for reducing the overall medical-imaging radiation exposure needed, as well as the potential for creating a dual drug, one containing both imaging and therapeutic applications via the inclusion of Re and Tc metal centres. The toxicity evaluation of any new compound is a major defining step as to whether a new compound has a commercial future or not and requires the take up of the frontline research into any pharmaceutical company’s research and development program (PhRMA, 2015 ▸; Eckstein, 2005 ▸; Tonkens, 2005 ▸; Torjesen, 2015 ▸).

The challenge for the chemist is how to localize the organo­metallic binding to the cancerous cells but not the normal tissue. Specific area injection is an obvious answer to this challenge, such as the use of heterogeneous ^188^Re-colloids for brachytherapy, which can be physically inserted at a site (Lepareur *et al.*, 2019 ▸). Another is the continued development of site-specific complexes (Liu, 2004 ▸). Agents that bind to a specific site in the biological organ with high concentration cause minimal damage to the surrounding tissue. This review clearly highlights an unusual commonality that supports the ideal of the latter suggestion. Of the 27 PDB entries containing rhenium listed in Table 1 ▸, 74% of these (*i.e.* 20 structures) show direct coordination of the metal to a histidine moiety via the imidazole group. This is a marked preference for one particular amino-acid residue, particularly when considering that crystallization conditions were markedly variable and involved various organometallic cores and oxidation states. Furthermore, 11 different proteins were analysed containing basically a full range of amino-acid residue types on their protein surfaces.

So, how might this whole field progress? Fundamentally there are some significant technical obstacles from a crystallographic aspect that must be addressed in future research.

Firstly, data have been extracted from the PDB over a broad time period spanning 20 years or more. Significant scientific and technology progress has occurred during this period, including in crystallization techniques, X-ray synchrotron/lasers/home sources, detectors, software developments, IUCr publication and validation requirements, CSD/wwPDB data submission and validation requirements *etc*. We also wish to emphasize the need of FAIR data principles (where FAIR data is findable, accessible, interoperable and reusable) in the field of macromolecular and chemical crystallography. The purpose of our review is not to criticize the authors of past articles we have referred to who did not have the tools available today. But we ask the question how can published data and tools currently made available to macromolecular crystallographers be interoperably utilized by scientists in disciplines other than the original purpose (Helliwell, 2019 ▸)? We have found that not all of our surveyed database deposits are ‘reusable’ since they do not contain the structure factors, a point we return to below.

Accurately examining multiple weak interactions should be extractable in either macromolecular or chemical crystallography and is crucial for drug development. A valuable tool provided by the CSD is *CSD-CrossMiner*. Care should be taken when viewing possible trends in protein–metal inter­actions, particularly when searching for *d*-block transition metals as carried out for this study. In such an organometallics review, we recommend a combined analysis is carried out via the stepwise-analyse-by-hand method, supported by the available search engines developed by the chemical and/or macromolecular crystallographic community to avoid missing any key information. Another future development that would be most useful is the availability of constructing space-volume calculations from small molecules and then being able to search for identical ‘space-volume pockets’ on proteins in their structures downloaded from the PDB. Thus, both electronic and steric factors could be examined either individually or collectively, a factor utilized in homogeneous catalysis research with calculations such as the Tolman cone angle (Tolman, 1977 ▸; Bilbrey *et al.*, 2013 ▸).

Thirdly, we see the need to extend our research, and the studies by others, to where the whole crystallography procedure is undertaken ideally at mammalian body temperature (37°C). This is quite challenging because the co-crystallization of the organometallic of interest with a protein should also be carried out at 37°C, not only the X-ray diffraction data collection. The crystallization conditions at room temperature (∼20 to 25°C) may not be the same at 37°C. Organometallic reaction-rate constants generally increase by a factor of two or three for each 10°C rise in temperature (Moore & Pearson, 1981 ▸). Such studies will assess whether the weaker occupancy binding sites would have increased metal occupation at body temperature or would migration to the dominant binding species (*i.e.* histidine) become more prominent? Also, could structure studies of proteins at variable temperatures increase our understanding of dynamic movements by examining the flexibility of side chains, or by the loss of water molecules (Helliwell, 2021 ▸; Tilton *et al.*, 1992 ▸, Sanchez *et al.*, 2019 ▸)? A recent review describes the practical aspects of preparing, acquiring and analysing X-ray crystallography data at room temperature, and sheds light on preconceived impracticalities that tend to deter most crystallographers from conducting routine room-temperature data collection at synchrotron sources (Fischer, 2021 ▸).

Fourthly, a fundamental difficulty of evaluating the precision of quite a number of PDB entries is the absence of their associated structure-factor files (such as in 1k4j, 1i53, 1jzi, 1r1c and 2fnw). Thus, the import of a particular PDB entry into *Coot* (Emsley & Cowtan, 2004 ▸) does not yield the difference electron-density map in such cases. To examine the difference electron-density map is vital for seeing features that are not the focus of the authors’ model (or original purpose) and specifically to check if there are any signs of structural disorder around the rhenium sites or possibly more weakly occupied metal sites. Specifically of relevance to this review is the question: could these disorders be eliminated and the rhenium compound harnessed to better advantage for a radiopharmaceutical biomedical application? If the structure factors are available to re-refine the model, this is not necessarily straightforward if the ligand restraints file is not available. The lack of interoperability of the PDB and the CSD in such a situation can be a considerable obstacle (Brink & Helliwell, 2019*b* ▸). Additional differences are observed between the authors indicating formal protein–metal bonding and protein–ligand interactions versus the RCSB NGL ligand viewer (Rose & Hildebrand, 2015 ▸; Rose *et al.*, 2017 ▸), which lists the weak interactions. The RCSB PDB clearly defines the criteria for the interaction types and the calculation parameters used. To gain greater clarity between possible discrepancies of this kind, it would be best to analyse the precision of each bond distance, factoring in the resolution of each PDB entry, diffraction data completeness *etc.*, to accurately determine which is a weak interaction and which is a formal bond.

And finally, it is important to note that absolute configuration is a key aspect affecting the chemistry of organo­metallic or inorganic compounds and therefore must be correctly illustrated or described. Many database entries interchangeably utilize SMILES or InChI notation when constructing 2D diagrams (or ligand CIFs for protein refinement). However, organometallic complexes are problematic to describe because their bonding scheme cannot fully be explained by valence-bond theory (David *et al.*, 2020 ▸). It is sometimes difficult (utilizing the notation) to clearly establish which atoms of the ligands are bound to the metal and to decide which bond-order scheme suits the specific organometallic compound the best (Quirós *et al.*, 2018 ▸). This often leads to ambiguity in representation when algorithms or automatic machine drawing tools are utilized (Heller *et al.*, 2015 ▸). It is therefore strongly recommended to always refer to the original publication and the PDB entry/CIF to view the correct organometallic configuration.

We hope that this topical review survey and descriptions of possible improvements to the methods will stimulate this important field for further, even enhanced, medical application.

## Figures and Tables

**Table:** (i) MAD or SAD phasing

PDB entry	3lya	6f9p	1hnu	1k4j	5k1j
Author	Eichinger *et al.* (2011 ▸)	Bastard *et al.* (2018 ▸)	Mursula *et al.* (2001 ▸)	Watson *et al.* (2002 ▸)	Ciccone *et al.* (2016 ▸)
Rhenium complex	K_2_ReCl_6_	K_2_ReCl_6_	Potassium perrhenate	Ammonium perrhenate	Rhenium tris-carbonyl pyta-C8 derivative
Method	X-ray diffraction	X-ray diffraction	X-ray diffraction	X-ray diffraction	X-ray diffraction
Protein macromolecule	Transcriptional activator CadC	L-Lysine 4-hy­droxy­lase	D3,D2-enoyl CoA isomerase ECI1	Acyl-homoserinelactone synthase (EsaI)	Human transthyretin
Protein:Re ratio	NS†	NS	1 m*M* potassium perrhenate (KReO_4_)	NS	NS
Crystallization conditions	Vapour-diffusion hanging drop. CadC_pd_ crystals transferred from mother liquor to 2.5*M* Li_2_SO_4_ to avoid the formation of metal–ammonia complexes. 30 m*M* K_2_ReCl_6_ and 1.7 *M* Li_2_SO_4_ cautiously added to the drop until final ReCl_6_^2−^ concentration reached 5 m*M*	24% PEG 3350, 0.2 *M* imidazole malate, 0.15 *M* Li_2_SO_4_, pH 7.0	Vapour-diffusion hanging drop in 0.1 *M* MES (pH 5.6), 5%(*w*/*v*) 1,4-dioxane and 1.4 *M* ammonium sulfate	Vapour-diffusion hanging-drop methods with PEG 4000, 2-propanol, MES, EDTA, β-mercapto­ethanol, NaN_3_, pH 6.1	21% PEG 4000 (PEG 4K) and 0.14 *M* imidazole malate, pH 6.0
Crystallization time	NS	NS	NS	NS	A few hours
Crystallization temperature (°C)	20	20	22	18	20
Cryoprotectant	2.5 *M* Li_2_SO_4_, 30%(*v*/*v*) glycerol	30% CM5, 26% PEG 3350, 0.2 *M* imidazole malate, pH 8, 0.15 *M* Li_2_SO_4_, 0.2 m*M* ReK_2_Cl_6_, 20 min soak	20%(*v*/*v*) ethyl­ene glycol	NS	Soaking for 10 min in cryoprotectant [40% SM2 (12.5% ethyl­ene glycol, 12.5% glycerol, 12.5% 1,2-propane­diol, 25% DMSO and 37.5% 1,4-dioxane) 25% PEG 8K] and 0.2 m*M* of the rhenium complex
No. of X-ray wavelengths and values (Å)	3 (1.17652, 1.17705 and 1.07813)	1 (1.1761)	4 (1.1765, 1.1773, 1.1697 and 1.1836)	4 (1.14, 1.1724, 1.1719 and 1.19)	1 (1.175919)
Space group	*P*6_1_22	*P*2_1_2_1_2_1_	*P*6_3_22	*P*4_3_	*P*2_1_2_1_2
Resolution (Å)	2.3	2.4	2.15	2.5	1.69
Protein–metal bonding‡	–	Glu178, His176 and His312	–	–	His88
Protein–metal complex weak interactions§	His344, Glu447, Thr229, Asp225 and Trp450	–	Asn239 and Leu126	Gln120, Lys161, Phe164, Cys85, Ser143, Ser119, Ser71, Tyr135 and Thr96	–
Notes	–	An unidentified blob with electron density less than 5σ is found at the rhenium metal site at what appears to be an open coordination position	Crystals were soaked for 4 h with 1 m*M* KReO_4_	No MTZ files available	Additional protein–rhenium interactions are possibly present (dependent on resolution) but have not been reported in the article. The Re atom sits in a pocket surrounded by Trp79, Thr75 and His90

**Table:** (ii) Electron transfer and/or electron tunnelling

PDB entry	6mjs	6mjt	6mjr	2i7o
Author	Takematsu *et al.* (2019 ▸)	Takematsu *et al.* (2019 ▸)	Takematsu *et al.* (2019 ▸)	Shih *et al.* (2008 ▸)
Rhenium complex†	*fac*-[Re(CO)_3_(dmp)]^+^	*fac*-[Re(CO)_3_(dmp)]^+^	*fac*-[Re(CO)_3_(dmp)]^+^	*fac*-[Re(CO)_3_(dmp)]^+^
Method	X-ray diffraction	X-ray diffraction	X-ray diffraction	X-ray diffraction
Protein	Azurin	Azurin	Azurin	Azurin
Protein:Re ratio	NS	NS	NS	NS
Crystallization conditions	Vapour-diffusion method. 0.1 *M* NaOAc, 120 m*M*/150 m*M* Li_2_SO_4_ and 48.6% PEG 400/27.7% PEG 8000 at pH 4.5	Vapour-diffusion method. 0.1 *M* NaOAc, 120 m*M*/150 m*M* Li_2_SO_4_, and 48.6% PEG 400/27.7% PEG 8000 at pH 4.5	Vapour-diffusion method. 0.1 *M* NaOAc, 120 m*M*/150 m*M* Li_2_SO_4_, and 48.6% PEG 400/27.7% PEG 8000 at pH 4.5	Vapour-diffusion hanging-drop method. 20–24% PEG 4000, 100 m*M* LiNO_3_, 100 m*M* citric acid, pH 3.2
Crystallization time	NS	NS	NS	NS
Crystallization temperature (°C)	21	21	21	24.85
Cryoprotectant	NS	NS	NS	NS
Number of X-ray wavelengths and values (Å)	1 (1.00)	1 (1.5406)	1 (1.5406)	1 (0.972)
Space group	*C*222_1_	*C*2	*P*2_1_2_1_2_1_	*I*222
Resolution (Å)	1.85	1.893	2.012	1.5
Protein–metal bonding‡	His126	His126	His126	His124
Protein–metal complex weak interactions§	Gln12, Ala119, Leu120, Gln107, Trp124 and Trp122	Trp124	Ala119, Gln107, Phe124 and Trp122	Trp122
Notes	–	–	–	–

**Table:** 

PDB entry	1i53	1jzi	1r1c	2fnw
Author	Di Bilio *et al.* (2001 ▸)	Crane *et al.* (2001 ▸)	Miller *et al.* (2003 ▸)	Blanco-Rodríguez *et al.* (2006 ▸)
Rhenium complex	[Re(CO)_3_(L)(H_2_O)](Otf) (L = phen or 4,7-Me_2_phen)	[Re^I^(CO)_3_(1,10-phen­anthroline)(His83)]	[Re^I^(CO)_3_(1,10-phen­anthroline)(His107)]	*fac*-[Re^I^(CO)_3_(1,10-phen­anthroline)(H_2_O)]CF_3_SO_3_
Method	X-ray diffraction	X-ray diffraction	X-ray diffraction	X-ray diffraction
Protein	Azurin	Azurin	Azurin	Azurin
Protein:Re ratio	NS	NS	NS	NS
Crystallization conditions	Crystals from 2 µl drops containing 26 mg ml^−1^ [Re(CO)_3_(4,7-Me_2_phen)(His107)]^+^AzCu_2_^+^ in 25 m*M* HEPES pH 7.5, equilibrated against a 500 µl reservoir containing 20% PEG 8000 and 100 m*M* in pH 8.0	Vapour-diffusion hanging drop. Crystals of metal-modified azurin grew from solutions of 20–30 mg ml^−1^ of protein in 40 m*M* imidazole and 2 m*M* NaCl (pH 7.2), mixed 50:50(*v*:*v*) with reservoirs of 100 m*M* imidazole (pH 6.0–8.0), 100 m*M* LiNO_3_, 6.25 m*M* CuCl_2_ and 25–38% PEG (4000–8000). ReAz crystals grew within a pH range of 6.0–8.0. The complex Cu^II^(imidazole)_4_(OH_2_)_2_ mediated crystal contacts	Vapour-diffusion sitting drop. Crystals grew from 2 µl drops made from equal volumes of 30 mg ml^−1^ ReAzCu^II^ in 25 m*M* HEPES pH 7.5 and reservoir. Drops were treated with 500 µl of reservoir containing 20% PEG 4000, 100 m*M* LiNO_3_ and 100 m*M* imidazole pH 7.0	Vapour-diffusion hanging drop. Crystals grew from 2 µl drops made from equal volumes of 30 mg ml^−1^ Re^I^-azurin in 25 m*M* HEPES pH 7.5 and reservoir. Drops were treated with 500 µl of reservoir containing 20% PEG 4000, 100 m*M* LiNO_3_ and 100 m*M* imidazole pH 7.0
Crystallization time	NS	NS	NS	NS
Crystallization temperature (°C)	21.85	20	25	25
Cryoprotectant	NS	NS	NS	NS
Number of X-ray wavelengths and values (Å)	1 (1.5418)	1 (1.08)	1 (0.9640)	1 (0.945)
Space group	*P*1	*C*2	*P*2_1_	*P*1
Resolution (Å)	1.80	1.62	1.9	1.4
Protein–metal bonding‡	His107	His83 and His283	His107	His83 (renamed to His309) and His109
Protein–metal complex weak interactions§	–	Val80 and Val280,	Ala53, His107 and Thr124	Lys122, Ala53, Lys322, Gln257 and Ala253
Notes	No MTZ files available	No MTZ files available	No MTZ files available	No MTZ files available

**Table:** 

PDB entry	4k9j	3ibo	2i7s
Author	Takematsu *et al.* (2013 ▸)	Blanco-Rodríguez *et al.* (2009 ▸)	Blanco-Rodríguez *et al.* (2009 ▸)
Rhenium complex	*fac*-[Re(CO)_3_(dmp)]^+^	*fac-*[Re(imidazole)(CO)_3_(1,10-phen­anthroline)]_2_SO_4_·4H_2_O	*fac-*[Re(imidazole)(CO)_3_(1,10-phen­anthroline)]_2_SO_4_·4H_2_O
Method	X-ray diffraction	X-ray diffraction	X-ray diffraction
Protein macromolecule	Azurin	Azurin	Azurin
Protein:Re ratio	1:1	NS	NS
Crystallization conditions	Vapour-diffusion sitting drop. Protein buffer: 40 m*M* imidazole, 2 m*M* NaCl. Reservoir: 100 m*M* imidazole, 100 m*M* LiNO_3_, 6.25 m*M* CuCl_2_, 27% PEG 4000, pH 7.4	Crystals grown from 2.5 µl drops made from 1 µl 30 mg ml^−1^ Re^I^-azurin in 25 m*M* HEPES pH 7.5, 0.5 µl saturated [Co(NH_3_)_5_Cl]Cl_2_, and 1 µl of reservoir solution. Drops were equilibrated against 500 µl reservoir containing 20% PEG 4000, 100 m*M* LiNO_3_ and 100 m*M* imidazole pH 7.0	Same conditions as 3ibo except that [Co(NH_3_)_5_Cl]Cl_2_ was not used
Crystallization time	After six months, seed crystals were harvested and used to grow larger crystals over four months	NS	NS
Crystallization temperature (°C)	24.85	24.85	24.85
Cryoprotectant	NS	NS	NS
Number of X-ray wavelengths and values (Å)	1 (0.9795)	1 (0.916)	1 (0.9480)
Space group	*F*222	*P*12_1_1	*P*12_1_1
Resolution (Å)	1.7	1.45	1.35
Protein–metal bonding‡	His126	His83, His109, His124 and His126	His124, His324, His524 and His724
Protein–metal complex weak interactions§	–	Thr124, Gln107 and Thr21	His124
Notes	–	–	–

**Table:** (iii) Medical applications

PDB entry	1b0q	3axf	3r26	3rj7
Author	Giblin *et al.* (1998 ▸)	Aryal *et al.* (2012 ▸)	Aryal *et al.* (2012 ▸)	Can *et al.* (2012 ▸)
Rhenium complex	[ReOCl_3_(Me_2_S)(OPPh_3_)]	Sodium perrhenate	Sodium perrhenate	[(CpCONH–CH_2_C_6_H_4_–SO_2_NH_2_)Re–(CO)_3_]
Method	Solution NMR	X-ray diffraction	X-ray diffraction	X-ray diffraction
Protein macromolecule	Cyclic α-MSH	ModA	ModA	hCAII
Protein:Re ratio	1:1	NS	NS	–
Crystallization conditions	1:1 peptide-exchange complex in 62% MeOH, pH 8–9 at 65–70°C for 1 h	Vapour-diffusion hanging drop. 26% PEG 8000, 0.1 *M* sodium acetate and 2.5 m*M* sodium perrhenate, pH 5.0 solution	Vapour-diffusion hanging drop. 26% PEG 8000, 0.1 *M* sodium acetate and 2.5 m*M* sodium perrhenate, pH 5.0 solution	2.4 *M* (NH_4_)_2_SO_4_, 0.1 *M* Tris–HCl at pH 7.5, 1 m*M* 4-(hy­droxy­mercuri) benzoic acid sodium salt and 5.1% vol. DMSO. hCAII crystals grown with the hanging-drop vapour-diffusion method and soaked with the respective inhibitor
Crystallization time	NA†	Two weeks	Two weeks	Four weeks at 298 K, or two weeks at 277 K then again four weeks at 298 K
Crystallization temperature (°C)	NA	25	25	25 or 4
Cryoprotectant	NA	10% glycerol, 27.5% PEG 8000 and 0.1 *M* sodium acetate (pH 4.5) for the wild-type protein; and 10% glycerol, 26% PEG 8000 and 0.1 *M* sodium acetate (pH 5.0) for the mutant	10% glycerol, 27.5% PEG 8000 and 0.1 *M* sodium acetate (pH 4.5) for the wild-type protein and 10% glycerol, 26% PEG 8000 and 0.1 *M* sodium acetate (pH 5.0) for the mutant	Paratone N
Number of X-ray wavelength and values (Å)	NA	1 (0.97857)	1 (0.97857)	1 (1.00)
Space group	NA	*P*2_1_2_1_2_1_	*P*322_1_	*P*12_1_1
Resolution (Å)	NA	2.0	1.7	1.2
Protein–metal bonding‡	Cys1, Cys7, and Tryp6	–	–	Zn atom found in the active site. No formal protein–rhenium bonds observed
Protein–metal complex weak interactions§	–	Pro124, Ala125, Tyr170, Ser12, Val152 and Ser39	Ser12, Ser39, Tyr170 and Val152	Thr199, Leu198, Val121, His15, Asp19, His4 and Asn11, Phe131, Pro202
Notes	In the PDB ligand view, the nitro­gen to rhenium inter­actions are not indicated as the data are sourced from NMR. From a coordination-chemistry perspective, these interactions should be reasonably strong and present as they complete the metal coordination sphere	–	–	The deprotonated nitro­gen of the rhenium aryl­sulfonamide complex terminus coordinates to the Zn atom

**Table:** 

PDB entry	3kam	3qng	3qe8
Author	Binkley *et al.* (2010 ▸, 2011 ▸)	Zobi & Spingler (2012 ▸)	Zobi & Spingler (2012 ▸)
Rhenium complex	*fac*-[Re(CO)_3_(H_2_O)_3_]Br	*fac*-[Re(CO)_3_(H_2_O)_3_]^+^ with L-serine	*fac*-[Re(CO)_3_(H_2_O)_3_]^+^ with imidazole
Method	X-ray diffraction	X-ray diffraction	X-ray diffraction
Protein macromolecule	Hen egg-white lysozyme (HEWL)	HEWL	HEWL
Protein:Re ratio	1:5	1:10	1:10
Crystallization conditions	0.05 *M* MES buffer (pH 5.5) and 0.8 *M* NaCl	0.05 *M* acetate buffer (pH 4.6) and 0.9 *M* NaCl	0.05 *M* acetate buffer (pH 4.6) and 0.9 *M* NaCl
Crystallization time	48–72 h	–	–
Crystallization temperature (°C)	19	25	25
Cryoprotectant	30% glycerol	25% glycerol	25% glycerol
Number of X-ray wavelengths and values (Å)	1 (1.54)	1 (1.00)	1 (1.00)
Space group	*P*4_3_2_1_2	*P*4_3_2_1_2	*P*2_1_2_1_2_1_
Resolution (Å)	1.59	1.55	1.49
Protein–metal bonding‡	His15	His15	His15
Protein–metal complex weak interactions§	–	Ile88, Asp87 and Thr89	Ile88
Notes	Soaking experiment yielded identical results	–	–

**Table:** 

PDB entry	5nbj	6ro3	6ro5
Author	Brink & Helliwell (2017 ▸)	Brink & Helliwell (2019*a* ▸)	Brink & Helliwell (2019*a* ▸)
Rhenium complex	*fac*-[Re(CO)_3_(H_2_O)_3_]^+^	*fac*-[Re(CO)_3_(H_2_O)_3_]^+^ and *fac*-[Re_4_(μ_3_-OH)_4_(CO)_12_]	*fac*-[Re(CO)_3_(H_2_O)_3_]^+^ and *fac*-[Re_4_(μ_3_-OH)_4_(CO)_12_]
Method	X-ray diffraction	X-ray diffraction	X-ray diffraction
Protein macromolecule	HEWL	HEWL	HEWL
Protein:Re ratio	1:30	1:30	1:30
Crystallization conditions	10% NaCl, 0.04 *M* sodium acetate (pH 4.7) and *fac*-[Et_4_N]_2_[Re(CO)_3_(Br)_3_] at 0.03 *M* in 1.4 ml water with DMSO [at 7.5%(*v*/*v*)]	10% NaCl, 0.04 *M* sodium acetate (pH 4.7) and *fac*-[Et_4_N]_2_[Re(CO)_3_(Br)_3_] at 0.03 *M* in 1.4 ml water with DMSO [at 7.5%(*v*/*v*)]	10% NaCl, 0.04 *M* sodium acetate (pH 4.7) and *fac*-[Et_4_N]_2_[Re(CO)_3_(Br)_3_] at 0.03 *M* in 1.4 ml water with DMSO [at 7.5%(*v*/*v*)]
Crystallization time	Approximately three weeks	Crystals were grown over approximately three weeks and left undisturbed for two years before collection	Crystals were grown over approximately three weeks and left undisturbed for a year before collection
Crystallization temperature (°C)	25	25	25
Cryoprotectant	Silicone oil	Silicone oil	Silicone oil
Number of X-ray wavelengths and values (Å)	2 (0.97625, 1.5418)	1 (0.9763)	1 (0.9763)
Space group	*P*4_3_2_1_2	*P*4_3_2_1_2	*P*2_1_2_1_2_1_
Resolution (Å)	1.266	1.03	1.68
Protein–metal bonding‡	His15, Leu129, Asp119, Asp18, Glu35, Asp52	His15, Asp119 and Glu7	His15, Asp52, Glu35, Asp119, Asp18 and Leu129
Protein–metal complex weak interactions§	Arg125, Glu7 and Arg61	Pro70, Arg5, Trp123, Lys33 and Asp87	Glu7, Lys1, Arg125, Asn46, Ile88, Thr89 and Asp87, Leu129
Notes	–	–	*fac*-[Re_4_(μ_3_-OH)_4_(CO)_12_] cluster is found as a non-coordinating entity

† Abbreviations: NS, not explicitly stated by the authors; NA, not applicable; dmp = 4,7-dimethyl-1,10-phenanthroline.

‡ Formal bonds observed between the protein residue and metal centre as indicated by the authors.

§ As viewed with RCSB PDB’s NGL ligand viewer and mentioned by the authors.
